# The role of NLRP3 inflammasome in the development of postoperative ileus and its molecular mechanism in male mice

**DOI:** 10.14814/phy2.71075

**Published:** 2026-08-20

**Authors:** Fan Zhang, Beicheng Sun, Hongqian Guo

**Affiliations:** ^1^ Department of Urology Nanjing Drum Tower Hospital Clinical College of Nanjing Medical University Nanjing China; ^2^ Department of General Surgery Nanjing Drum Tower Hospital Clinical College of Nanjing Medical University Nanjing China

**Keywords:** cell pyrosis, inflammatory reaction, MCC950, NLRP3 inflammasome, oxidative stress, postoperative ileus

## Abstract

This study investigated the roles of the NLRP3 inflammasome in postoperative ileus (POI) pathogenesis. Male BALB/c mice were randomly divided into sham, POI, and MCC950 (NLRP3 inhibitor) groups. Intestinal motility was assessed 24 h postoperatively. RNA‐seq was performed. Histopathological changes were evaluated through H&E and immunofluorescence; CAT and POD activities in the intrinsic muscle layer were detected; Western blot was used to detect NLRP3, GSDMD‐N, cleaved caspase‐1, and p‐NF‐κB p65 expressions; ELISA was used to detect blood IL‐1β, TNF‐α, IL‐6, and IL‐10 levels. RNA‐seq analysis showed 152 differential genes in the POI group, including NLRP3. In intestinal tissue of the POI mice, NLRP3 expression and expressions of GSDMD‐N, cleaved caspase‐1, and p‐NF‐κB p65 increased. IL‐1β, TNF‐α, IL‐6, and IL‐10 levels increased, while CAT, POD, and SOD activities decreased. The postoperative small intestine advancement rate of POI mice was reduced. After MCC950 intervention, the pathological damage to intestinal tissue and the intestinal function were restored, and the small intestine advancement rate was improved. Meanwhile, MCC950 effectively inhibited the activation of the NLRP3 inflammasome. NLRP3 inflammasome activation drives the pathogenesis of POI and MCC950 can effectively alleviate intestinal injury.

## 1 INTRODUCTION

Postoperative ileus (POI) is a common condition after abdominal surgery, which reflects the deceleration or complete cessation of intestinal movement. This complication is very common, affecting 10%–25% of patients after abdominal surgery (Buscail & Deraison, 2022; Chapman et al., 2018). Its characteristics are transient gastrointestinal motility disorders, leading to delayed gastric emptying, accumulation of gas and fluid, bloating, and prolonged hospitalization (Shogan et al., 2020; Wang, Stakenborg, & Boeckxstaens, 2025) The pathophysiology of POI is multifactorial, involving neurogenic, inflammatory, and pharmacological mechanisms. Intestinal surgery triggers an inflammatory response in the outer layer of the intestinal muscle layer, manifested as the recruitment and activation of white blood cells, which release pro‐inflammatory cytokines and nitric oxide, directly inhibiting smooth muscle contractility (Buscail & Deraison, 2022; Stein et al., 2018; Urbanek et al., 2024). Unlike mechanical intestinal obstruction, POI is not caused by physical blockage of the intestinal lumen, but by functional peristaltic inhibition driven by inflammation and neural reflexes. Therefore, we need to further study the relevant mechanisms of the occurrence and development of intestinal obstruction to find better diagnosis and treatment methods for early diagnosis and treatment of intestinal obstruction.

Inflammatory bodies are large multi‐protein complexes within cells that play an important role in innate immunity. They can recognize pathogen‐associated molecular patterns (PAMPs) and damage‐associated molecular patterns (DAMPs) and activate downstream inflammatory responses. The NLRP3 inflammasome is one of the most extensively studied inflammasomes, consisting of the NLRP3 protein, adaptor protein ASC, and effector protein pro‐caspase‐1. Activated NLRP3 inflammasome can recruit and activate caspase‐1, which can cleave gasdermin D (GSDMD), triggering cell pyroptosis and promoting the maturation and release of inflammatory factors such as IL‐1β and IL‐18 (Huang et al., 2021; Yan et al., 2021).

At present, there is no research on the relationship between NLRP3 inflammasomes and intestinal obstruction. However, the relationship between NLRP3 and intestinal diseases has been widely reported. Studies have shown that transplantation of intestinal microorganisms from NLRP3 knockout mice is likely to become a new strategy for the treatment of depression (Li, Fang, et al., 2022). The role of NLRP3 inflammasome in inflammatory bowel disease (IBD) has also been reported. There is evidence that the polymorphism of NLRP3 gene contributes to the low expression of NLRP3 inflammasome and affects the genetic susceptibility of IBD (Chen et al., 2019). Therefore, based on the above, we speculate that NLRP3 inflammasomes may play a critical role in the progression of POI; on the other hand, inhibiting the level of NLRP3 may also help promote the recovery of POI. Therefore, this study will focus on the role of NLRP3 inflammatory bodies in the occurrence and development of POI and its related mechanisms.

## 2 MATERIALS AND METHODS

### 2.1 Animals and grouping

Male BALB/c mice were raised in a specific pathogen‐free (SPF) environment under controlled conditions (22°C, 50%–60% humidity, 12‐h light/dark cycle) with free access to food and water. Mice are fed standard rodent feed (#XTC01WJ‐002, Jiangsu Xiehe Pharmaceutical Bioengineering Co., Ltd., China). Adaptive feeding was conducted for 1 week before the experiment. The mice were then randomly assigned to three groups: control group, POI group, and POI model + MCC950 (#HY‐12815A, MedChemExpress, USA) treatment group (10 mg/kg) (Gauthier et al., 2025). A priori power analysis (G*Power, two‐sided *t*‐test, *α* = 0.05, 1−*β* = 0.80) using effect sizes estimated from preliminary data (or from a similar study) indicated that a minimum of 6 mice per group was required. To allow for up to 30% attrition due to surgical complications, we included 10 mice per group, resulting in a total of 30 mice across three groups. All experimental procedures were performed in accordance with the ethical guidelines and management standards for laboratory animals of Nanjing Drum Tower Hospital.

### 2.2 Establishment of POI animal model

All mice were fasted for 12 h before operation but with free access to water. Thirty minutes before the surgery, the mice were placed in the animal operating room for preparation. All surgical procedures were performed under sterile conditions. Anesthesia was induced and maintained using inhaled isoflurane (He et al., 2025). After confirming the absence of a response to painful stimulation, the mice were fixed on the operation board, the abdominal skin was disinfected with iodophor, and the sterile hole towel was paved. A midline abdominal incision of approximately 1.5 cm was made, and the skin, subcutaneous tissue, and rectus abdominis muscle were incised layer by layer to expose the peritoneum. Then the peritoneum was carefully incised, with the incision slightly smaller than that of the abdominal wall. The small intestine was gently exteriorized from the abdominal cavity using a sterile cotton swab and placed on a sterile towel. Then a sterile cotton swab dipped in pre‐cooled phosphate‐buffered saline (#C0221A, Beyotime, pH 7.4) was used to gently wipe both sides of the small intestine and the mesenteric side from the distal duodenum to the lower end of the cecum, avoiding direct wiping of the mesenteric side to prevent vessel rupture and bleeding. Wiping was performed three times in the same direction, with a total duration of 8–10 min, until obvious congestion and edema of the small intestine were observed (Dai, 2017). After wiping, the small intestine was slowly returned to the abdominal cavity in the order from duodenum to cecum to avoid twisting or knotting. Then the peritoneum was continuously sutured with 5–0 absorbable suture, and the abdominal muscles and skin were sutured intermittently. During the whole operation, the operating table temperature was maintained at 28°C–30°C to prevent hypothermia. After the operation, the mice were resuscitated in a 32°C constant temperature incubator in the supine position. After their autonomous activities were restored, the temperature of the incubator was gradually reduced to 30°C and 28°C every 2 h, and finally maintained at 26°C. The mice were fasted but allowed free access to water for 24 h postoperatively. The modeling time, body weight changes, and general condition of the mice were recorded. Among the 30 mice used in the study, 3 mice died immediately during or after surgery (mortality rate: 10%): 2 mice in the POI group, 1 mouse in the POI + MCC950 group, due to major bleeding or anesthesia related complications. These mice were excluded from the analysis and new mice were added to maintain *n* = 10 per group. The inclusion criteria for successful POI modeling are: (1) obvious intestinal congestion and edema observed during abdominal exploration 24 h after surgery; (2) The small intestine propulsion rate is 50% lower than the average of the control group (Gauthier et al., 2025).

### 2.3 Dosage regimen

MCC950 (# HY‐12815A, MedChemExpress, USA) was dissolved in a solvent (0.9% physiological saline containing 5% DMSO). POI + MCC950 groups of mice were intraperitoneally injected with MCC950 at doses of 10 mg/kg once a day for 30 min before surgery and three consecutive days after surgery. The control group and POI group were injected with equal volumes of solvent.

### 2.4 Small intestine propulsion rate test

To evaluate the motility of the small intestine, a small intestine transport test was conducted 24 h after surgery. Each mouse was given 0.2 mL of black charcoal powder. After applying charcoal for 20 min, the mice were euthanized using the carbon dioxide method, and the entire small intestine (from the pylorus to the cecum) was carefully removed and placed on a moist, flat surface without tightening. The total length of the small intestine and the distance traveled by charcoal powder from the pylorus were measured. The propulsion rate was calculated as follows: distance traveled by charcoal/total length of small intestine × 100%.

### 2.5 Collection and processing of the samples

#### 2.5.1 Blood sample

After all mice were anesthetized with isoflurane inhalation, the abdominal cavity was opened, the abdominal aorta was found, and the blood collection needle was inserted into the artery. The needle tail was placed into an anticoagulant vacuum blood collection tube for blood collection and then centrifuged at 3000 r/min for 15 min. The supernatant was collected using a pipette and placed in a cryovial, and finally stored in a liquid nitrogen tank.

#### 2.5.2 Tissue samples

After blood collection, mice were euthanized using the carbon dioxide (CO_2_) inhalation method. Specifically, mice were placed in a clean, transparent euthanasia chamber (non‐precharged) and CO_2_ was introduced from a compressed gas cylinder at a gradual fill rate of 10%–20% of the chamber volume per minute (approximately 2–3 L/min). Following loss of consciousness, the CO_2_ flow rate was maintained for at least 5 min after respiratory arrest. Death of the mice was confirmed by assessing the absence of heartbeat, respiration, and pedal withdrawal reflex, followed by cervical dislocation as a secondary physical method to ensure death, consistent with the AVMA Guidelines for the Euthanasia of Animals (2020 Edition). Immediately after the confirmation of death, the abdominal cavity was quickly opened to collect the small intestine tissue.

### 2.6 RNA isolation and transcriptome analysis

RNA was extracted with Trizol (#15596026, invitrogen, USA), followed by quantification of concentration and purity using Nanodrop 2000. All samples showed OD260/280 ratios within the range of 1.8–2.0. Agilent 2100 biological analyzer was used to detect the integrity of the RNA (RIN value >7.0). Qualified samples were used for subsequent database building. Oligo (DT) magnetic beads (#S1419S, NEB, USA) were used to enrich the mRNAs and fragment them at high temperature. cDNA first strands were generated using fragmented mRNA as the templates, and then the second strand was synthesized, and the terminal repair, A‐tail and junction connection were performed. Hieffngs® DNA selection beans (#12601ES56, Yeasen, China) were used for fragment selection and PCR amplification to construct the library. Quality inspection confirmation was conducted through Qubit 2.0 fluorescence analyzer and Agilent 2100 biological analyzer, 150 bp double ended sequencing was performed on MGI dnbseq‐t7 platform. Raw sequencing data were quality‐controlled and filtered by fastp to generate clean reads, which were then aligned to the mouse reference genome (grcm38) using HISAT2. Gene expression was quantified in FPKM, and differential analysis was performed with edgeR. Following screening (FDR <0.05, |log_2_FC| ≥ 1), the resulting differential genes were analyzed for GO functional enrichment and KEGG pathways. GO enrichment analysis was performed using the DAVID database (https://david.ncifcrf.gov, version 2021). KEGG pathway analysis was conducted using KEGG Mapper (https://www.genome.jp/kegg/mapper/, version 2.5). We constructed a PPI network by interrogating the STRING database (https://string‐db.org, version 11.5). The sequencing analysis included 3 POI mice and 3 controls.

### 2.7 Histopathological analysis

After graded ethanol dehydration and xylene clearing, the tissue samples were embedded in paraffin. Subsequently, after obtaining the tissue sections (4 μm) using a Leica RM2245 microtome, they were baked (60°C, 2 h), dewaxed with xylene, and rehydrated using an ethanol gradient according to standard procedures. Hematoxylin staining was performed for 5 min, after which the slides were rinsed under running water, differentiated briefly in 1% acid‐alcohol, and blued in running water. The samples were stained by eosin for 2 min and rinsed with running water. After gradient ethanol dehydration and xylene transparency, the neutral gum was used to seal the slices. The intestinal tissue structure was observed under the light microscope, and the mucosal integrity, inflammatory cell infiltration, and edema degree were also evaluated. Each experiment was repeated 3 times.

### 2.8 Detection of catalase (CAT), peroxidase (POD) and superoxide dismutase (SOD) activities in mouse intestinal muscle

The small intestine tissue sample frozen at −80°C was taken, and pre‐cooled RIPA lysis buffer containing protease inhibitors (#04693159001, Roche, Switzerland) was added. The tissue was then homogenized using a tissue homogenizer, centrifuged at 12,000 × g for 15 min at 4°C, and the supernatant was then collected. The total protein concentration in the supernatant of each sample was determined using a BCA protein quantification kit (#P0012, Beyotime, China). The activities of catalase (CAT), peroxidase (POD), and superoxide dismutase (SOD) were measured using commercially available kits (Beyotime, CAT: Beyotime, #S0051; POD: Beyotime, #S0119; SOD: Beyotime, #S0088) according to the instructions provided by the manufacturer. Each experiment was repeated 10 times.

### 2.9 Immunofluorescence staining

After dewaxing and hydration of paraffin sections, antigen repair was carried out: antigen retrieval was performed by heating in sodium citrate buffer (pH 6.0) to boiling in a microwave, maintaining heat for 15 min, and then cooling naturally to room temperature. This was followed by permeabilization with 0.3% Triton X‐100 (10 min) and blocking with 5% BSA (30 min, # P0007, Beyotime). Rabbit anti‐NLRP3 primary antibody (1:200, # 19771‐1‐AP, Proteintech, USA) was applied overnight at 4°C. The second day, FITC detection antibody (1:500, ab6717, Abcam, UK) was added dropwise and incubated at room temperature in the dark for 1 h. DAPI was stained for 5 min and sealed with anti‐fluorescence quenching agent (#P0126, Beyotime, China). Use Nikon Eclipse Ci‐L fluorescence microscope (equipped with DS‐Fi3 camera and NIS Elements v5.21 software) to capture images. Objective lenses: 4 × (NA 0.10), 10 × (NA 0.25), 20 × (NA 0.45), 40 × (NA 0.75). FITC detection wavelength 490/525 nm, DAPI detection wavelength 358/461 nm. Quantitative analysis of fluorescence expression levels using ImageJ. Each experiment was repeated 3 times.

### 2.10 Western blot

The protein concentration of each sample was determined using the BCA method, and 30 μg of protein per sample was loaded for electrophoresis. Depending on the molecular weight of the target proteins, gels of different concentrations were used: an 8% gel for proteins between 80 and 150 kDa, a 10% gel for proteins between 16 and 70 kDa, and a 12% gel for proteins between 12 and 45 kDa. Electrophoresis was performed in two stages: samples were first stacked in a concentrating gel at 80 V for 30 min, then separated in a resolving gel at 120 V for 1 h. After electrophoresis, the membrane was blocked in 5% bovine serum albumin (BSA) blocking solution for 1 h, washed with TBST, and then incubated overnight at 4°C with primary antibodies (NLRP3, 1:1000, Proteintech, #19771‐1‐AP; GSDMD‐N, 1:1000, Abcam, #ab215203; cleaved caspase‐1, 1:800, Cell Signaling Technology, #89332; p‐NF‐κB p65, 1:1000, Cell Signaling Technology, #3033; GAPDH, 1:1000, Proteintech, #60004‐1‐Ig). The membrane was then washed three times with TBST and incubated with an HRP‐linked secondary antibody (#7074, Cell Signaling Technology, USA) at room temperature for 60 min. After additional washes, chemiluminescence (#WBKLS0500, Millipore, USA) detection was performed using a developer. Each experiment was repeated 3 times.

### 2.11 Enzyme linked immunosorbent assay (ELISA)

Plasma samples were collected from mice, centrifuged at 3000 rpm for 15 min at 4°C, and the plasma was separated and stored at −80°C until analysis. The concentrations of inflammatory cytokines, including interleukin‐1β (IL‐1β, #88‐7013‐88, Thermo Fisher Scientific, USA), tumor necrosis factor‐α (TNF‐α, #88‐7324‐88, Thermo Fisher Scientific, USA), interleukin‐6 (IL‐6, #88‐7064‐88, Thermo Fisher Scientific, USA), and interleukin‐10 (IL‐10, #88‐7105‐88, Thermo Fisher Scientific, USA), were measured using commercially available kits. All procedures were performed strictly according to the manufacturer's instructions. Each experiment was repeated 10 times.

### 2.12 Statistics

The data were expressed as mean ± standard deviation (mean ± SD). All data were first subjected to normality testing using the Shapiro Wilk test. For data that conform to normal distribution, parameter testing is used: one‐way ANOVA is used for comparison between multiple groups, followed by Tukey HSD test for pairwise comparison; compare between two groups using Student's *t*‐test. For data that do not follow a normal distribution, Kruskal–Wallis test is used for intergroup comparisons, followed by Dunn post hoc test. All tests were independently repeated at least three times. The statistical significance is set to *p* < 0.05.

## 3 RESULTS

### 3.1 Successful establishment of POI model and analysis of transcriptome characteristics

**FIGURE 1:**
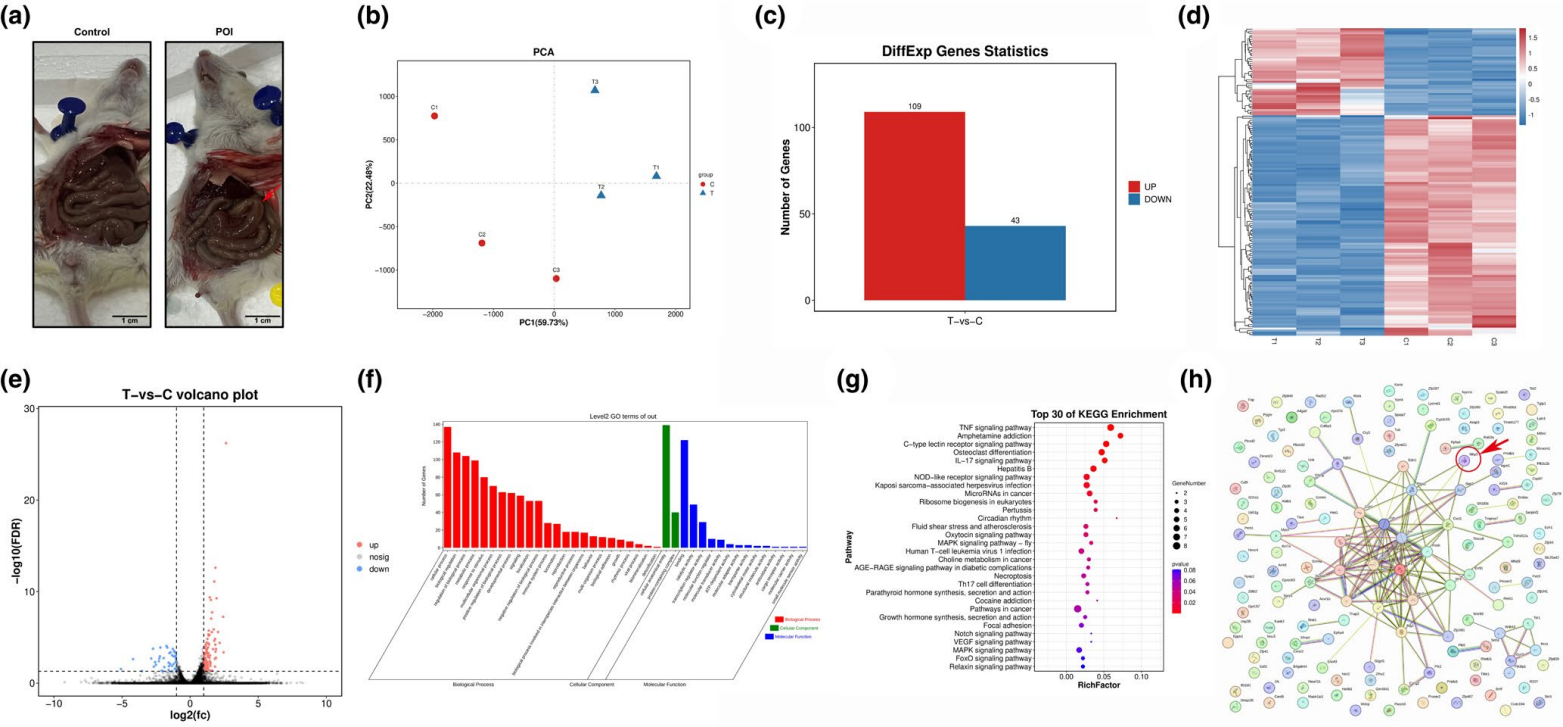
Establishment of POI model and analysis of transcriptome characteristics. (a) Establishment of mouse POI model; (b) PCA results analysis; (c) up and down regulated genes; (d) heat plot; (e) volcano plot; (f) go analysis; (g) KEGG analysis; (h) PPI.

First, POI mice were modeled. 24 h after operation, it was found that the small intestine of POI group mice was markedly dilated, the intestinal wall was congested and edematous, and the intestinal cavity was filled with pale yellow effusion (Figure 1a), indicating that we successfully established the POI model of mice. So as to explore the molecular mechanism of POI at the genome‐wide level, we conducted RNA‐seq using small intestinal tissues of control versus POI model mice. Principal component analysis (PCA) showed that the two groups of samples were completely separated, and there was no overlap between them, indicating that POI caused significant changes in the transcriptome of intestinal tissue (Figure 1b). Through differential gene analysis, we identified 152 genes with significant changes in expression, including 109 upregulated and 43 downregulated genes (Figure 1c–e). Go functional enrichment analysis showed that among the items related to biological processes, differentially expressed genes may be involved in cellular processes, biological regulation, response to stimuli and biological processes of the immune system; among the items related to cell components, the differentially expressed genes may participate in the components such as cell anatomical entities and protein complexes; in terms of molecular functions, differentially expressed genes may be involved in binding activity, catalytic activity, small molecule sensor activity and other functions (Figure 1f). Enrichment analysis using KEGG pathways indicated that the DEGs were mainly concentrated in exhibited significant clustering in multiple signaling pathways closely related to inflammation, such as TNF, NOD like receptors, IL‐17, and MAPK (Figure 1g). The protein–protein interaction (PPI) network analysis showed that NLRP3 was identified as one of the central nodes in the network, directly or indirectly interacting with various inflammatory factors and signaling molecules, suggesting its potential importance in the POI inflammation regulatory network (Figure 1h).

### 3.2 NLRP3 inflammasome is specifically activated in POI

**FIGURE 2:**
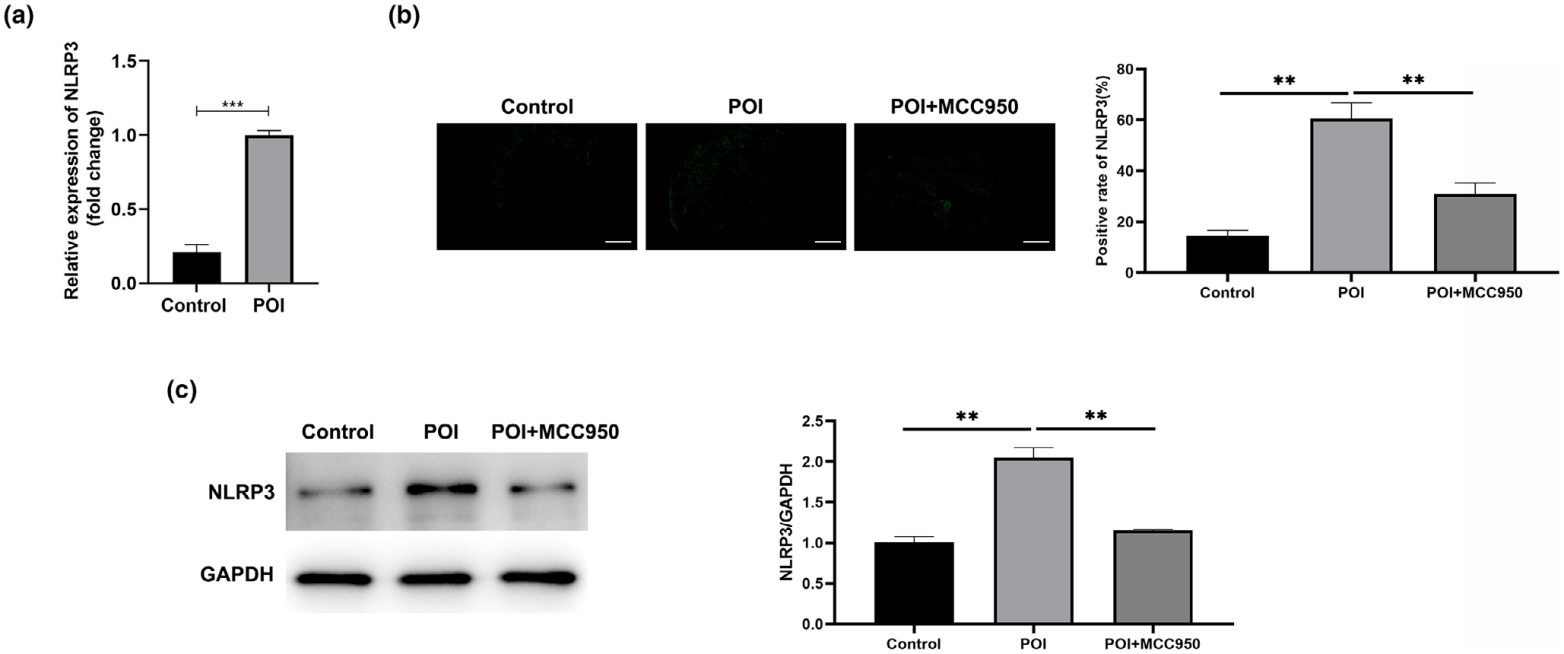
Expression of NLRP3 inflammasome in POI. (a) Relative expression of NLRP3; (b) fluorescence staining of NLRP3 in intestinal tissues of animals in each group; (c) detection of NLRP3 protein in intestinal tissues of animals in each group. **p < 0.01, ***p < 0.001.

Analysis of the significantly altered genes revealed that NLRP3 exhibited significant up‐regulation in the POI model (Figure 2a, *p* < 0.001). To verify the RNA SEQ results, we further treated the POI model by inhibiting MCC950 with NLRP3. Immunofluorescence staining showed that compared with the control group, the POI model group showed an increase in NLRP3 signal, while after MCC950 intervention, the amount of NLRP3 in the intestinal tissue of mice showed a decreasing trend (Figure 2b, *p* < 0.01). The WB results further confirmed the findings of immunofluorescence staining mentioned above. The level of NLRP3 in the small intestine tissue of POI model mice was higher than that in the sham operation control group; In contrast, mice intervened with MCC950 showed a decrease in intestinal NLRP3 expression compared to the POI model group (Figure 2c, *p* < 0.01). These observations establish that the intestinal NLRP3 inflammasome is significantly activated during POI and MCC950 can effectively inhibit its expression.

### 3.3 NLRP3 inhibitor MCC950 can improve intestinal inflammatory injury of POI

**FIGURE 3:**
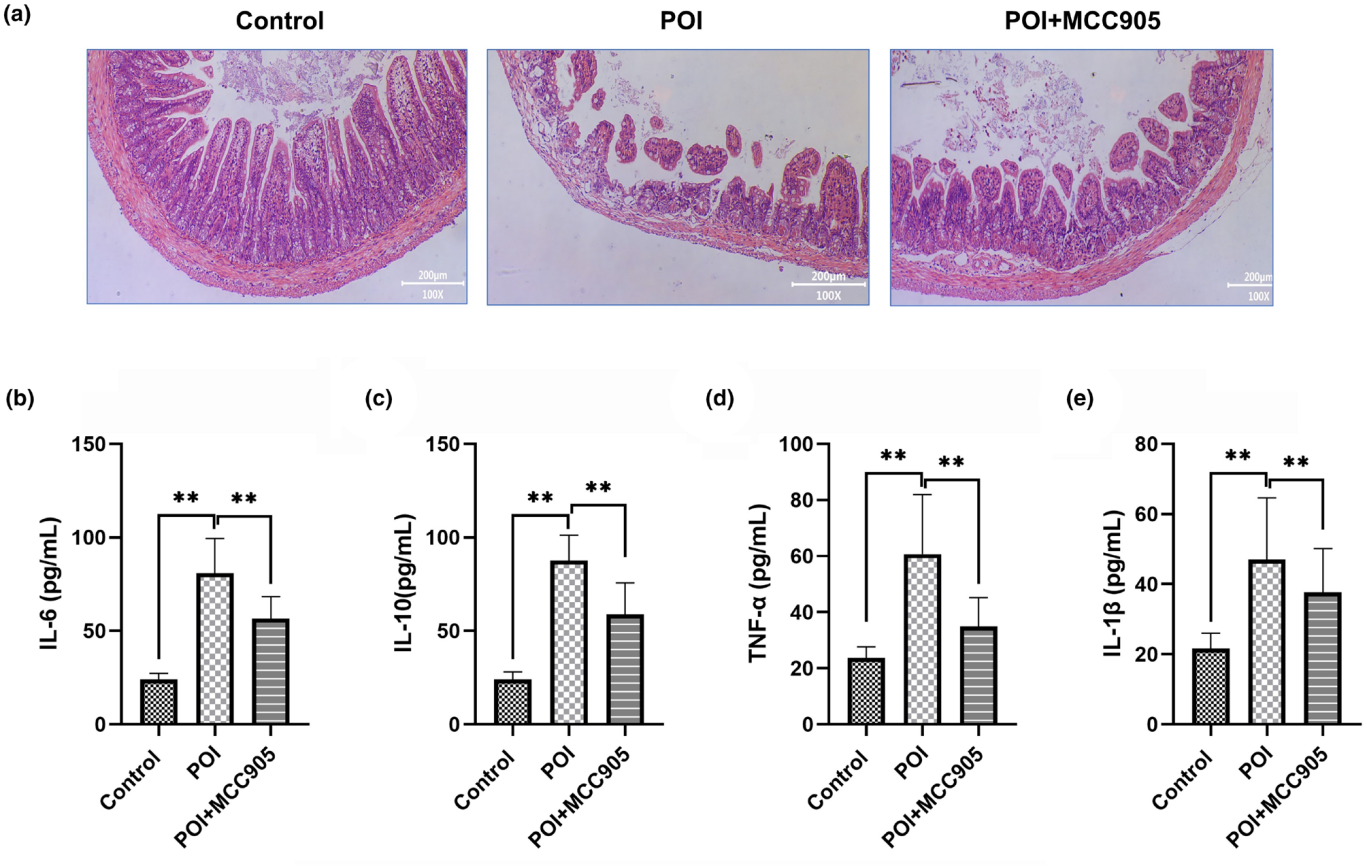
Effect of NLRP3 inhibitor MCC950 on intestinal inflammatory injury of POI. (a) Intestinal H&E staining; (b) ELISA was used to detect the release of IL‐6; (c) the release of IL‐10 was detected by ELISA; (d) the release of TNF‐α was detected by ELISA; (e) the release of IL‐1β was detected by ELISA. **p < 0.01.

Subsequently, we evaluated the pathological consequences of NLRP3 activation and the intervention effect of MCC950. H&E staining showed that the small intestinal mucosal structure of mice in the POI group was severely damaged, which was characterized by villi breaking off and a large number of inflammatory cell infiltrations. MCC950 treatment significantly improved the tissue morphology and reduced inflammatory cell infiltration (Figure 3a). ELISA detected significant increases in IL‐1β, TNF‐α, IL‐6, and IL‐10 within the POI group, and MCC950 treatment could effectively reduce the concentrations of these factors (Figure 3b–e, *p* < 0.01). In addition, to directly evaluate intestinal motility, we measured small intestine transport rate using charcoal meal tests. As shown in Table 1, compared with the control group, the small intestine propulsion rate of the POI group was significantly reduced (*p* < 0.01), indicating severe inhibition of peristalsis. MCC950 treatment significantly improved the advancement rate (compared to the POI group, *p* < 0.01), indicating that intestinal motility has been restored.

**TABLE 1:** Small intestine propulsion rate test in each group of mice.

Group	Coal powder propulsion distance (cm)	Small intestine length (cm)	Promotion rate (%)	P (promotion rate, vs. POI model group)
Control	28.05 ± 3.35	38.10 ± 2.51	73.39 ± 4.04	0.00
POI	11.21 ± 2.52	38.30 ± 3.23	28.95 ± 4.19	
POI + MCC950	19.86 ± 3.13	38.21 ± 3.23	51.70 ± 3.83	0.00

### 3.4 NLRP3 inhibitor MCC950 can restore the imbalance of oxidative stress in POI

**FIGURE 4:**
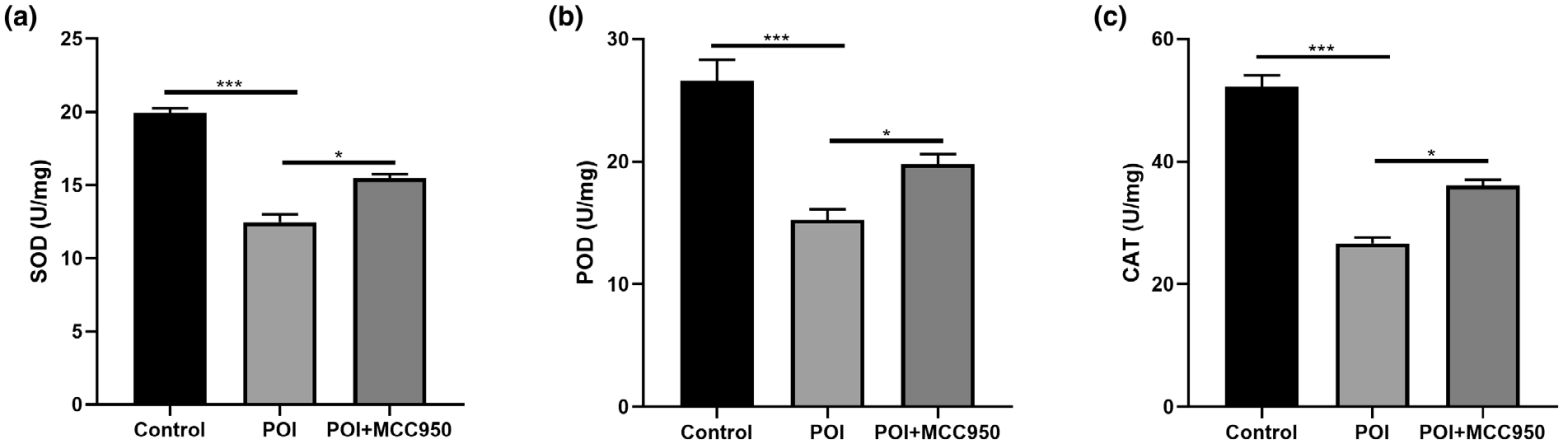
Effect of NLRP3 inhibitor MCC950 on oxidative stress imbalance in POI. (a) Catalase (CAT) activity detection; (b) the activity of peroxidase (POD) was detected; (c) superoxide dismutase (SOD) activity was detected. *p < 0.05, ***p < 0.001.

Oxidative stress is closely intertwined with inflammation. We detected the activities of three major antioxidant enzymes in the homogenate of the small intestine. The experimental data showed that the activities of CAT, POD, and SOD in the intestines of POI model group mice significantly decreased. After the intervention of MCC950, the activity of the three antioxidant enzymes mentioned above increased to varying degrees in intestinal tissue (Figure 4a–c, *p* < 0.05). This indicates that the activation of NLRP3 in POI is associated with severe oxidative stress, and inhibition of NLRP3 helps to restore the antioxidant capacity of the body.

### 3.5 NLRP3 inhibitor MCC950 inhibits POI cell pyroptosis

**FIGURE 5:**
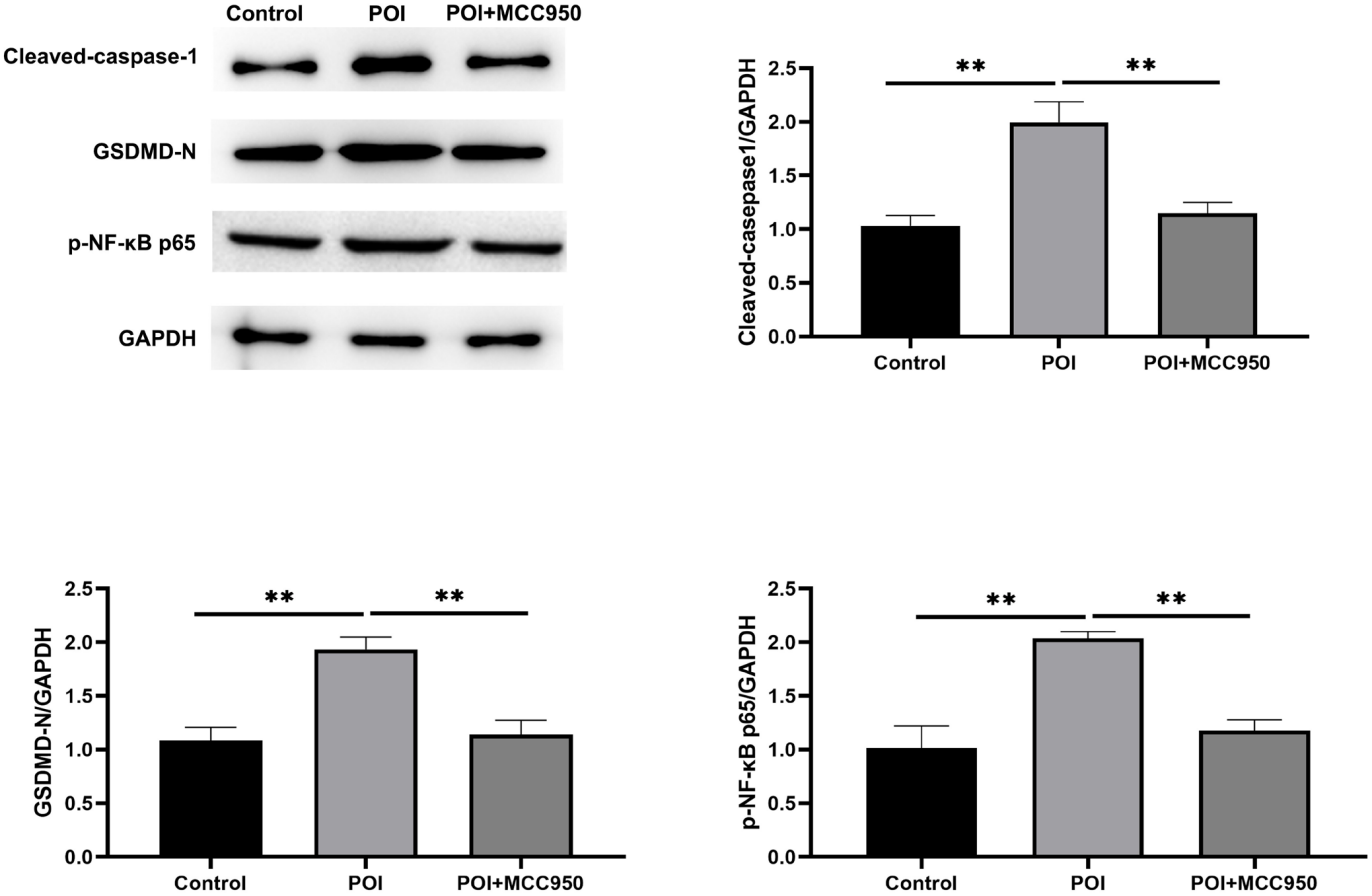
Effect of NLRP3 inhibitor MCC950 on pyroptosis of POI cells. WB was used to detect the protein expressions of cleaved caspase1, GSDMD‐N and p‐NF‐κ B p65. **p < 0.01.

To clarify the pathway of action of NLRP3 in POI, we detected its downstream key proteins. WB analysis found that the expression of GSDMD‐N, cleaved caspase‐1, and small intestinal p‐NF‐κB p65 was increased in POI mice relative to controls, and MCC950 intervention could effectively reverse this trend (Figure 5, *p* < 0.01). These findings imply the notion that NLRP3 inflammasome is activated during POI, which promotes caspase‐1 activation and GSDMD‐N production, may induce cell pyroptosis; at the same time, the NF‐κB signaling pathway was activated to amplify the inflammatory response. MCC950 can effectively inhibit this series of cascade reactions.

## 4 DISCUSSION

The common symptoms of postoperative ileus (POI) are general hypomotility of the gastrointestinal tract and delayed gastric emptying. POI is a common pathological condition after abdominal intestinal surgery, and it may also occur after other types of surgery (Rodriguez‐Padilla et al., 2021; Wattchow et al., 2021). The treatment of POI mainly focuses on promoting intestinal peristalsis (such as using lidocaine) and anti‐inflammatory (such as using nonsteroidal anti‐inflammatory drugs). However, patients often need to fast after surgery, which severely limits the use of drugs, and the effectiveness of existing therapies still requires more clinical evidence to support (Domen et al., 2021; van Beekum et al., 2021). This study found that the activation of the NLRP3 inflammasome plays a key role in the occurrence and development of POI. Surgical trauma, intestinal ischemia–reperfusion, and other factors can induce intestinal inflammatory reactions, which in turn activate the NLRP3 inflammasome, leading to the release of inflammatory factors such as IL‐1β, exacerbating intestinal damage and dysfunction. Therefore, in‐depth exploration of the inflammatory mechanisms related to POI, especially the role of the NLRP3 inflammasome, is crucial for developing effective prevention and treatment strategies.

The inflammasome functions as a multiprotein complex, including nod like receptors (NLRs) and non NLR proteins, which signal through classical and non‐classical signaling pathways. There are many kinds of NLRs in cells, which aggregate in response to specific stimuli (Shi et al., 2024; Zong et al., 2023). Recent studies have begun to reveal the role of inflammasomes in the pathophysiology of POI. For example, Hupa et al.'s 2019 study suggests that AIM2 inflammasome may be one of the key drivers of IL‐1β release in POI, and this release occurs in a biphasic manner, with AIM2 playing a critical role in the later stages. These findings suggest that the inflammatory cascade of POI may involve the synergistic or independent action of multiple inflammasomes (Hupa et al., 2019). In addition, previous studies have shown that blocking IL‐1β signaling (using anakinins) can significantly improve postoperative inflammation and intestinal obstruction in mice, further emphasizing the therapeutic potential of inflammasome IL‐1β axis in POI (Stoffels et al., 2014). As the most extensively studied inflammasome associated with various inflammatory diseases, NLRP3 has been widely reported in intestinal diseases such as inflammatory bowel disease (IBD). Studies have shown that the polymorphism of the NLRP3 gene affects the genetic susceptibility of IBD (Yoganathan et al., 2021). In the intestinal ischemia/reperfusion injury model, the metabolite indole‐3‐carboxaldehyde of Bifidobacterium longum can inhibit the activation of NLRP3 inflammasome in macrophages by blocking HDAC3 (Miao et al., 2025). However, the specific contribution and molecular mechanism of NLRP3 inflammasome in the occurrence and development of POI, especially through the regulation of cell pyroptosis and oxidative stress pathways, still need to be further elucidated.

In the project, RNA‐seq technology helped elucidate the transcriptome of small intestine tissues of POI model mice and control mice, in order to reveal the changes of key gene expression in the development of POI and its possible biological processes and signaling pathways at the genome‐wide level. Gene expression profiles differed significantly between the POI and control groups, as revealed by transcriptome analysis, with a total of 109 upregulated and 43 downregulated genes identified. Functional enrichment analysis further revealed that these DEGs showed predominant association with biological processes such as inflammation, immune regulation, cell pyroptosis, and oxidative stress, and significantly aggregate in signaling pathways closely related to inflammation and immunity, such as TNF, NOD like receptors, IL‐17, etc. Notably, the manifestation of NLRP3 was significantly up‐regulated in the POI group, which confirmed the activation of the NLRP3 inflammasome in the pathogenesis of POI at the transcriptional level. The innate immune receptor NLRP3 orchestrates a downstream cascade involving caspase‐1 activation, which subsequently cleaves and activates pro‐inflammatory cytokines, notably IL‐1β and IL‐18, to drive inflammation and pyroptotic cell death. The upregulation of NLRP3 was observed in the POI model, suggesting that NLRP3 may mediate the pathogenesis of POI by driving the local inflammatory response of the intestine and promoting neutrophil infiltration.

For further verification, we measured NLRP3 abundance in the intestinal tissue of model group mice by WB method. Experimental data found that the expression of NLRP3 in the intestinal tract of model animals was upregulated, which is consistent with previous theoretical speculation; at the same time, the level of NLRP3 in the intestinal tract of POI mice treated with NLRP3 inhibitor MCC950 was significantly decreased. Therefore, NLRP3 is potentially implicated in the pathogenesis of POI.

MCC950 (also known as CRID3) is a potent and selective NLRP3 inflammasome small molecule inhibitor (Li, Guan, et al., 2022). It can specifically bind to the NACHT domain of NLRP3, blocking its ATPase activity, thereby inhibiting NLRP3 oligomerization and inflammasome assembly (Coll et al., 2019). At the highest concentration of 10 mg/kg used in this study, MCC950 has been shown to achieve sufficient plasma and tissue concentrations to effectively inhibit NLRP3 dependent IL‐1β production (Li et al., 2023; Ren et al., 2024).

It is generally believed that POI is due to the continuous stage of postoperative intestinal motility decline caused by intestinal treatment, which is caused by the performance of inflammatory mechanism (Mazzotta et al., 2023; Schneider et al., 2021; Yang et al., 2021). More specifically, delayed intestinal transport may be related to the activation of local macrophages and the induction of neutrophil infiltration in the small intestine muscle layer after surgery (Enderes et al., 2020). Elevated serum levels of cytokines such as IL‐1β, TNF‐α, IL‐6, and IL‐10 were observed in the POI group, which is consistent with the general understanding of intestinal inflammation; however, MCC950 significantly reduced these inflammatory markers in the treatment group. The results of this study are consistent with existing research on the efficacy of MCC950 in other intestinal inflammation models, further confirming the key role of NLRP3 inflammasome in regulating intestinal inflammation (Perera et al., 2018) Therefore, our results suggest that the changes of these cytokines in the blood of intestinal tissue after intestinal obstruction can be alleviated after treatment with NLRP3 inhibitor MCC950.

The pathological mechanism of POI is accompanied by oxidative stress. Research has shown that blocking the activation of NLRP3 can directly enhance the Nrf2 signaling pathway and alleviate oxidative stress damage (Cheng et al., 2022). Inflammation of the mucosal muscle layer stimulates α‐2 adrenoceptor, which can play a role in intestinal obstruction by increasing inducible nitric oxide synthase and activating cyclooxygenase‐2 (Chen et al., 2022; Lu et al., 2020). It was found that during the treatment of POI, surgery activated macrophages in the muscularis externa, increased the secretion of inflammatory factors including TNF‐α and IL‐1, and promoted the release of nitric oxide (no) and prostaglandins. Thus inhibiting the contraction of smooth muscle. In addition, the local release of intestinal neurotransmitters, especially substance P (SP), can directly activate mast cells and then cause neuroinflammation (Bakhshalizadeh et al., 2023; Yan et al., 2022). This study found that the activities of CAT, POD, and SOD in the intestinal tissue of the POI group were significantly decreased, and the activities of CAT, POD, and SOD in the intestinal tissue of the POI group were significantly increased after MCC950 treatment. Collectively, the data indicated that the oxidative stress reaction in the POI model suggested that the activity of antioxidant enzymes in the intestinal tissue of intestinal obstruction after operation was significantly reduced, and the treatment with NLRP3 inhibitor MCC950 could effectively alleviate the oxidative stress reaction caused by POI.

The inflammatory response of POI largely depends on various signaling pathways between cells, including activation of a series of kinases, phosphorylation, activation of transcription factors, transcription factors entering the nucleus to start transcription of pro‐inflammatory genes and so on (Peters et al., 2020). Inflammatory corpuscles are large multi protein complexes in cells and orchestrate innate immunity. Inflammatory corpuscles can detect pathogen‐ and damage‐associated molecular patterns (PAMPs/DAMPs), and initiate downstream immune responses. The activation of the above modes will lead to the activation of NF‐κB pathway, leading to the transcription up regulation of inflammatory body related components. In this study, the significant increase in p‐NF‐κB p65 levels in the intestinal tissue of POI model mice also confirmed the activation of this pathway. In a lung inflammation model, Wang et al. found that indole‐3‐aldehyde can activate aromatic hydrocarbon receptors (AhR), inhibit histone deacetylase (HDAC) activity, and downregulate the NF‐κB/NLRP3 signaling pathway, ultimately reducing inflammation (Wang, Tao, et al., 2025). Although the study was conducted in lung diseases, the AhR/HDAC/NF‐κB/NLRP3 regulatory axis revealed provides a new perspective for understanding the activation mechanism of NLRP3 in POI. In the sterile inflammatory environment of POI, damage associated molecular patterns (DAMPs) may also promote the phosphorylation of NF‐κB by affecting the activity of AhR or HDAC, thereby initiating the transcription and assembly of NLRP3 inflammasomes. After activation of the NF‐κB pathway, the inflammasome adapter protein ASC is recruited to NLRP3 and subsequently interacts with caspase‐1, triggering its activation (Shogan et al., 2020). Activated caspase‐1 can catalyze the maturation of inflammatory cytokines. In addition, infiltrating inflammatory cells can regulate intestinal smooth muscle function by secreting pro‐inflammatory and chemokines (Parnasa et al., 2021). These findings indicate that the expression of GSDMD‐N, cleaved caspase‐1, and p‐NF‐κB p65 is significantly increased in the intestinal tissue of the POI group, while the expression of GSDMD‐N, cleaved caspase‐1, and p‐NF‐κB p65 is significantly decreased in the intestinal tissue of the POI model treated with MCC950. The mechanism by which MCC950 reduces cell pyroptosis and inflammatory cytokine release by inhibiting the Caspase‐1/GSDMD‐N axis has been confirmed in various inflammatory models (Li et al., 2025; Xu et al., 2025). Similar effects were observed in the POI model in this study, further supporting the important role of pyroptosis in the pathological process of POI.

The innovation of this study is mainly reflected in the following three aspects. Firstly, the specificity of the disease context: Previous studies have mostly focused on the role of NLRP3 in chronic intestinal inflammation, while POI is an acute, sterile postoperative inflammatory response triggered mainly by mechanical damage and the release of risk related molecular patterns caused by surgical procedures. This study is the first to extend the pathological function of NLRP3 from chronic intestinal inflammation to acute postoperative intestinal motility disorders. Secondly, the specificity of the mechanism pathway: This study not only validated the activation of the classic inflammatory pathway NLRP3/caspase‐1/IL‐1β, but also revealed the cleavage activation of the pyroptosis executing protein GSDMD‐N and the phosphorylation enhancement of the NF‐κB signaling pathway, suggesting that NLRP3 participates in POI intestinal injury through a dual mechanism of inducing cell pyroptosis and amplifying the inflammatory cascade reaction. Thirdly, the specificity of intervention strategies: This study used the highly selective NLRP3 small molecule inhibitor MCC950 for intervention, which confirmed the therapeutic value of targeting NLRP3 from a functional perspective and provided experimental evidence for the potential application of NLRP3 inhibitors in the treatment of postoperative complications.

There are still several limitations to this study. Firstly, only male mice were selected as experimental subjects, aiming to eliminate the interference of estrogen fluctuations on inflammatory responses during the female estrus cycle. However, estrogen can regulate macrophage function and NLRP3 expression, which may affect the severity and treatment response of POI. Therefore, whether the conclusions of this study can be extended to the female population needs further verification. Secondly, this study only collected samples from animals euthanized 24 h after surgery. Although this time point can capture the acute inflammation peak of POI, the pathological process of human POI usually lasts for several days and involves multiple stages such as inflammation resolution, tissue repair, and intestinal motility recovery. Single point observation cannot evaluate the long‐term evolution process of POI and the delayed side effects of MCC950. Thirdly, no negative control with only secondary antibodies was set up in immunofluorescence detection, and no intestinal spontaneous fluorescence quenching treatment was performed. The intestinal tissue, especially the mucosal muscle layer, is rich in collagen fibers and exhibits significant spontaneous fluorescence within the FITC spectral range, which may affect the quantitative accuracy of NLRP3 fluorescence signal. Fourthly, this study has limitations in transcriptome analysis: the number of differentially expressed genes detected is relatively small (152), which may be related to insufficient sequencing depth and small sample size (3 per group). Subsequent research should expand sample size and optimize sequencing depth. Fifthly, this study observed a decrease in antioxidant enzyme activity in the intestinal tissue of POI group mice, suggesting that oxidative stress may be involved in the pathological and physiological processes of POI. However, the causal relationship between oxidative stress and NLRP3 activation is currently uncertain. Future research could consider using antioxidant interventions to observe their effects on NLRP3 activation and POI severity. Sixth, it is unclear in which cells (macrophages, epithelial cells, neurons, or smooth muscle cells) NLRP3 is activated. Seventh, this study is a correlation description and does not clarify the upstream activation mechanisms (DAMPs, ROS, ion disturbances) and how the pyroptosis pathway leads to motility disorders. Further in‐depth research is needed in the future.

In conclusion, our study systematically explored the relationship between NLRP3 inflammasomes and the occurrence and development of POI for the first time. Through transcriptome analysis, protein expression detection and pharmacological intervention, it was confirmed that NLRP3 inflammasomes were specifically activated in POI, and were involved in the course of the disease of POI by promoting inflammatory response, oxidative stress and cell death. At the same time, studies have confirmed that specific inhibitor MCC950 can effectively alleviate POI related pathological changes. This work establishes a new mechanistic insight for the potential role of NLRP3 in POI, and also provides a new direction and potential drug targets for the treatment of POI.

## AUTHOR CONTRIBUTIONS

**Fan Zhang:** Conceptualization; formal analysis; investigation; methodology; visualization. **Beicheng Sun:** Conceptualization; project administration; resources; supervision. **Hongqian Guo:** Conceptualization; funding acquisition; resources; supervision.

## FUNDING INFORMATION

This work was supported by the Jiangsu Province Capability Improvement Project through Science, Technology and Education, Jiangsu Provincial Medical Key Discipline (Laboratory) Cultivation Unit (JSDW202221).

## CONFLICT OF INTEREST STATEMENT

The authors declare no competing interests.

## ETHICS STATEMENT

All animal experiments were reviewed and approved by the Experimental Animal and Welfare Ethics Committee of Nanjing Drum Tower Hospital (approval No. 2023AE01035). Animals were handled in strict accordance with the national and institutional guidelines for the care and use of laboratory animals.

## Data Availability

The RNA‐seq data generated in this study have been deposited in the Gene Expression Omnibus (GEO) database under accession number GSEXXXXX (to be provided upon acceptance). Other data supporting the findings of this study are available from the corresponding author upon reasonable request.
